# *IRF6* rs2235375 single nucleotide polymorphism is associated with isolated non-syndromic cleft palate but not with cleft lip with or without palate in South Indian population^☆^

**DOI:** 10.1016/j.bjorl.2017.05.011

**Published:** 2017-06-26

**Authors:** Venkatesh Babu Gurramkonda, Altaf Hussain Syed, Jyotsna Murthy, Bhaskar V.K.S. Lakkakula

**Affiliations:** a Sri Ramachandra University, Department of Biomedical Sciences, Chennai, India; b Sri Ramachandra University, Department of Plastic Surgery, Chennai, India; c Sickle Cell Institute Chhattisgarh, Raipur, India

**Keywords:** IRF6, Orofacial clefts, NSCL/P, SNP, IRF6, Fendas orofaciais, NSCL/P, SNP

## Abstract

**Introduction:**

Transcription factors are very diverse family of proteins involved in activating or repressing the transcription of a gene at a given time. Several studies using animal models demonstrated the role of transcription factor genes in craniofacial development.

**Objective:**

We aimed to investigate the association of IRF6 intron-6 polymorphism in the non-syndromic cleft lip with or without palate in a South Indian population.

**Methods:**

173 unrelated nonsyndromic cleft lip with or without cleft palate patients and 176 controls without clefts patients were genotyped for *IRF6* rs2235375 variant by allele-specific amplification using the KASPar single nucleotide polymorphism genotyping system. The association between interferon regulatory factor-6 gene intron-6 dbSNP208032210:g.G>C (rs2235375) single nucleotide polymorphism and non-syndromic cleft lip with or without palate risk was investigated by chi-square test.

**Results:**

There were significant differences in genotype or allele frequencies of rs2235375 single nucleotide polymorphism between controls and cases with non-syndromic cleft lip with or without palate. *IRF6* rs2235375 variant was significantly associated with increased risk of non-syndromic cleft lip with or without palate in co-dominant, dominant (OR: 1.19; 95% CI 1.03–2.51; *p* = 0.034) and allelic models (OR: 1.40; 95% CI 1.04–1.90; *p* = 0.028). When subset analysis was applied significantly increased risk was observed in cleft palate only group (OR dominant: 4.33; 95% CI 1.44–12.97; *p* = 0.005).

**Conclusion:**

These results suggest that IRF6 rs2235375 SNP play a major role in the pathogenesis and risk of developing non-syndromic cleft lip with or without palate.

## Resumo

**Introdução:**

Fatores de transcrição constituem uma família de proteínas muito diversa envolvida na ativação ou repressão da transcrição de um gene, em um determinado momento. Vários estudos usando modelos animais demonstraram o papel dos genes do fator de transcrição no desenvolvimento craniofacial.

**Objetivo:**

Nosso objetivo foi investigar a associação do polimorfismo IRF6 intron-6 na fenda labial não sindrômica com ou sem fenda palatina em uma população do sul da Índia.

**Método:**

Um total de 173 pacientes com fenda labial não sindrômica com ou sem fenda palatina e 176 controles sem fendas foram genotipados para a variante IRF6 rs2235375 por amplificação alelo-específica utilizando o sistema KASPar de genotipagem de polimorfismo de nucleotídeo único. A associação entre o polimorfismo de nucleotídeo único Fator 6 Regulatório do Interferon(IRF6) intron-6 dbSNP208032210:g.G>C (rs2235375) e o risco de fenda labial não sindrômica com ou sem fenda palatina foi investigado pelo teste qui-quadrado.

**Resultadoss:**

Houve diferenças significativas nas frequências de genótipos ou alelos do rs2235375 SNP entre controles e casos com fenda labial não sindrômica com ou sem fenda palatina. A variante IRF6 rs2235375 foi significativamente associada ao aumento do risco de fenda labial não sindrômica com ou sem fenda palatina em modelos codominantes, dominantes (OR: 1,19; IC 95%: 1,03-2,51; *p* = 0,034) e alélicos (OR: 1,40; IC 95%: 1,04-1,90; *p* = 0,028). Quando a análise do subgrupo foi realizada, um risco significativamente aumentado foi observado no grupo Fenda Palatina Isolada (OR dominante: 4,33; IC 95%: 1,44-12,97; *p* = 0,005).

**Conclusões:**

Esses resultados sugerem que o polimorfismo de nucleotídeo único IRF6 rs2235375 desempenha um papel importante na patogênese e risco de desenvolvimento de fenda labial não sindrômica com ou sem fenda palatina.

## Introduction

Transcription factors are very diverse family of protein involved in activating or repressing the transcription of a gene at a given time. During development transcription factors are responsible for dictating the fate of individual cells. Several lines of evidence have demonstrated the role of transcription factor genes in craniofacial development and also the variants in these genes played a crucial role in the aetiology of NSCL/P.^1^, ^2^ Interferon regulatory factor-6 (IRF6) is a transcription factor that codes helix-turn-helix DNA binding domain and less conserved protein binding domain. In humans, nine IRFs were reported and the amino acid sequence composition analysis showed 89% similarity between IRF6 and IRF5, which plays a role in interferon activation and tumour suppression.^3^ In situ hybridization of mouse embryos demonstrated that IRF6 is highly expressed in the medial edges of the paired palatal shelves immediately before, and during, their fusion. IRF6 expression was also detected in hair follicles, palatal rugae, tooth germ, thyroglossal duct, external genitalia and on the skin throughout the body.^4^ Recent research has shown that IRF6 mutant mice exhibit a hyper-proliferative epidermis that fails to undergo terminal differentiation, which leads to multiple epithelial adhesions that can occlude the oral cavity and result in cleft palate.^5^

Interferon regulatory factor-6 (IRF6) is located in chromosome 1q32.2 position and is one of the candidate genes associated with both syndromic and non-syndromic forms of clefts.^6^, ^7^, ^8^ Further, several GWAS studies identified *IRF6* as an associated locus for oral clefts.^9^, ^10^ This gene contains 10 exons and among them exon 1, 2, and 10 are noncoding. IRF6 encodes protein contains total 517 amino acids and contains an N-terminal winged-helix DNA-binding domain and a C-terminal SMIR (Smad-interferon regulatory factor-binding) domain. Studies on humans showed that common alleles in IRF6 were associated with NSCL/P in different populations.^11^

Our previous study indicated that Haplotypes of two *IRF6* gene polymorphisms are associated with NSCL/P.^12^ In this study, we extend our previous work to evaluate the association between *IRF6* Intron-6 dbSNP208032210:g.G>C (rs2235375) single nucleotide polymorphism with non-syndromic cleft lip with or without palate risk in South Indian Population.

## Methods

Institution Ethics Committee of Sri Ramachandra University, Chennai, India, has approved the study design (Ref: IEC-NI/II/OCT/25/60 dated 12.01.2012). Informed consent was collected from parent or legal guardian when the subject was a minor. The study was comprised of 176 NSCL/P cases (77 female and 99 male) and 173 controls (77 female and 96 male). Study participants were recruited from Sri Ramachandra cleft and craniofacial centre, Sri Ramachandra University, Chennai, India. Two surgeons independently assessed cleft phenotype and cases with mental retardation or any other anomalies were excluded from the study. Of the 176 NSCL/P cases, 104 have cleft lip with cleft palate (CL/P; 76 unilateral and 28 bilateral), 40 have Cleft Lip Only (CLO) and 29 have cleft palate only (CPO). Both CLO and CPO cases are unilateral and none of the patients have affected relatives. Age and gender matched subjects without family history of clefting were recruited as controls. From each subjects 3 mL peripheral blood sample was collected and DNA was extracted using a standard procedure.^13^ Genotyping of the IRF6 rs2235375 SNP was performed by KBioscience by using KASPar chemistry.^14^, ^15^ SNP genotyping through KASPar chemistry involves competitive allele-specific PCR using FRET quencher cassette oligos (Table 1). Based on the fluorescence obtained, allele call data were viewed graphically as a scatter plot using the SNPViewer (http://www.lgcgenomics.com). Hardy–Weinberg Equilibrium (HWE) was assessed in both cases and controls groups by using chi-square test. Allele frequencies were estimated by the gene counting method.^16^ Comparison of genotype and allele frequencies among cases and the control groups were analyzed by the chi-square test. Odds ratio and 95% confidence intervals were calculated using wild type genotypes or allele as reference group.

**Table 1:** Primers used for genotyping *IRF6* rs2235375.

Primers^a^	Sequence^b^
Allele 1 (FAM)	5′-GTAAGTGAGACTTTATCTTTCTTGCTG-3′
Allele 2 (HEX)	5′-GTAAGTGAGACTTTATCTTTCTTGCTC-3′
Common reverse	5′-GAAAGCAGGACAGGAAAGAGTCTATAATA-3′

a Primers corresponding to different alleles were labelled with FAM and HEX fluorescent dyes (KBiosciences).

b Polymorphic bases are underlined.

## Results

The distribution of the *IRF6* rs2235375 variant genotypes and alleles in both cases and NSCL/P groups are presented in Table 2. The proportions of genotypes were 30.1% GG, 49.7% GC, 20.2% CC in cases and 40.9% GG, 44.3% GC, 14.8% CC in controls. The C allele frequency was 45.1% in cases and 36.9% in controls. The frequencies of *IRF6* rs2235375 genotype was distributed according to the Hardy–Weinberg equilibrium among the controls (*p* = 0.519). Significant difference in allele frequencies were found between control and the NSCL/P groups (Table 3). OR and 95% CI were calculated to assess the relative risk of oral clefts by comparing genotype frequencies of cases and controls in co-dominant, dominant as well as in allelic models (Table 3). Significantly increased NSCL/P risk was found for homozygous genotype (CC vs. GG; OR = 1.86; 95% CI 1.0–3.47; *p* = 0.047). Increased NSCL/P risk was also found under dominant (GC + CC vs. GG; OR = 1.19; 95% CI 1.03–2.51; *p* = 0.034) and allelic models (C vs. G; OR = 1.40; 95% CI 1.04–1.90; *p* = 0.028). In subgroup analysis, *IRF6* rs2235375 variant showed significantly increased risk in CPO under three different models (Table 3).

**Table 2:** Genotype distribution and allele frequencies of the *IRF6* rs2235375 SNP in cleft lip and palate.

	Control (%)	Overall clefts (%)	CL/P (%)	CPO (%)
*Genotype distribution*				
GG	72 (40.9)	52 (30.1)	48 (33.3)	4 (13.8)
GC	78 (44.3)	86 (49.7)	69 (47.9)	17 (58.6)
CC	26 (14.8)	35 (20.2)	27 (18.8)	8 (27.6)

*Allele frequency*				
G allele	222 (63.1)	190 (54.9)	165 (57.3)	25 (43.1)
C allele	130 (36.9)	156 (45.1)	123 (42.7)	33 (56.9)

*Test for HWE*				
Chi-square	0.417	0.003	0.062	0.996
*p*-value	0.519	0.959	0.803	0.318

CL/P, cleft lip with cleft palate; CPO, cleft palate only; HWE, Hardy–Weinberg equilibrium.

**Table 3:** Results of association tests with *IRF6* rs2235375 SNP in cleft lip and palate.

*IRF6* rs2235375	OR (95% CI)	*p*-value
*Overall clefts*		
GG	Reference	0.085^a^
GC	1.53 (0.95–2.44)	0.077
CC	1.86 (1.00–3.47)	0.047
GC + CC vs. GG	1.19 (1.03–2.51)	0.034
G allele	Reference	
C allele	1.40 (1.04–1.90)	0.028

*CL/P*		
GG	Reference	0.334^a^
GC	1.33 (0.81–2.16)	0.225
CC	1.56 (0.81–2.99)	0.18
GC + CC vs. GG	1.38 (0.88–2.19)	0.163
G allele	Reference	
C allele	1.27 (0.93–1.75)	0.137

*CPO*		
GG	Reference	0.014^a^
GC	3.92 (1.26–12.21)	0.021
CC	5.54 (1.54–19.95)	0.004
GC + CC vs. GG	4.33 (1.44–12.97)	0.005
G allele	Reference	
C allele	2.25 (1.28–3.96)	0.003

IRF6, interferon regulatory factor-6; OR, odds ratio; CI, confidence interval.

a P Value from a chi-square (df = 2).

## Discussion

Our findings clearly demonstrate that *IRF6* rs2235375 play a predominant role in the development of NSCL/P. This study provides confirmatory evidence for the variants which have been reported earlier, to be associated with NSCL/P. During the embryonic development, the role of IRF6 has been identified, but its regulatory function remains unclear.^5^, ^17^ At the time of palate fusion, degradation of the Medial Edge Epithelium (MEE) takes place and increased IRF6 expression was observed in MEE during this process.^18^, ^19^ Developmental expression patterns of IRF6 orthologues in mouse and chick revealed presence of IRF6 expression in the ectoderm fusion forming the upper lip and primary palate in both mouse and chick, but only in the developing secondary palate of the mouse.^20^

Meta-analysis of 20 published case-control studies demonstrated that the rs2235371 and rs642961 are antagonistic to each other in attributing the risk of NSCL/P.^21^ Allele “A” of a functional polymorphism (rs2235371; 820G > A) contributed to an increased risk of NSCL/P in Chinese population.^22^ On the contrary, “G” allele was over-transmitted in few studies.^11^ Few haplotype based analyses confirmed that the rs2235371 was associated with NSCL/P.^23^, ^24^, ^25^ In contrast to this rs2235371 SNP failed to show an association with oral clefts in some populations.^26^, ^27^, ^28^, ^29^ We previously showed that the *IRF6* rs2235371 G allele is over-transmitted in Indian clefts patients, but 820GG genotype contributed to minor risk only.^30^ There were few association studies available for SNP rs642961 and the results were inconsistent. Significant association between rs642961 and NSCL/P was observed.^26^, ^31^, ^32^, ^33^, ^34^ In contrast to the above mentioned studies, no association was reported by several studies.^29^, ^35^, ^36^, ^37^

Genome-wide association studies and their follow-up replication studies established *IRF6* as one of the candidate genes for the pathogenesis of NSCL/P.^38^ The polymorphism analyzed in this study (rs2235375) is located in the intron-6 of *IRF6*. This polymorphism showed the positive association between this variant and NSCL/P in several populations such as Italian,^7^ European-Americans,^25^ Norwegian,^39^ Chilean,^28^ Chinese^40^ and Brazilian.^41^

## Conclusion

In conclusion, the results of the present study indicate that *IRF6* rs2235375 polymorphism is associated with NSCL/P in South Indian population.

## Conflicts of interest

The authors declare no conflicts of interest.
